# Exploring the Regulatory Landscape of Dementia: Insights from Non-Coding RNAs

**DOI:** 10.3390/ijms25116190

**Published:** 2024-06-04

**Authors:** Jung-min Kim, Woo Ryung Kim, Eun Gyung Park, Du Hyeong Lee, Yun Ju Lee, Hae Jin Shin, Hyeon-su Jeong, Hyun-Young Roh, Heui-Soo Kim

**Affiliations:** 1 Department of Integrated Biological Sciences, Pusan National University, Busan 46241, Republic of Korea; jmk95@naver.com (J.-m.K.); dnfud647@pusan.ac.kr (W.R.K.); ehdtodt@pusan.ac.kr (E.G.P.); doo2080@naver.com (D.H.L.); lsg5821@naver.com (Y.J.L.); 0705haejin@naver.com (H.J.S.); tbd97@pusan.ac.kr (H.-s.J.); 2 Institute of Systems Biology, Pusan National University, Busan 46241, Republic of Korea; susan9416@naver.com; 3 Department of Biological Sciences, College of Natural Sciences, Pusan National University, Busan 46241, Republic of Korea

**Keywords:** microRNA, dementia, Alzheimer’s disease, frontotemporal dementia, Lewy body dementia, vascular dementia, long non-coding RNA, circular RNA, competing endogenous RNA

## Abstract

Dementia, a multifaceted neurological syndrome characterized by cognitive decline, poses significant challenges to daily functioning. The main causes of dementia, including Alzheimer’s disease (AD), frontotemporal dementia (FTD), Lewy body dementia (LBD), and vascular dementia (VD), have different symptoms and etiologies. Genetic regulators, specifically non-coding RNAs (ncRNAs) such as microRNAs (miRNAs), long non-coding RNAs (lncRNAs), and circular RNAs (circRNAs), are known to play important roles in dementia pathogenesis. MiRNAs, small non-coding RNAs, regulate gene expression by binding to the 3′ untranslated regions of target messenger RNAs (mRNAs), while lncRNAs and circRNAs act as molecular sponges for miRNAs, thereby regulating gene expression. The emerging concept of competing endogenous RNA (ceRNA) interactions, involving lncRNAs and circRNAs as competitors for miRNA binding, has gained attention as potential biomarkers and therapeutic targets in dementia-related disorders. This review explores the regulatory roles of ncRNAs, particularly miRNAs, and the intricate dynamics of ceRNA interactions, providing insights into dementia pathogenesis and potential therapeutic avenues.

## 1. Introduction

Dementia is a complex neurological syndrome that can significantly disrupt daily life through memory loss and impaired thinking and decision-making processes [1,2]. The incidence of dementia is on the rise, particularly before the age of 65, highlighting the importance of new strategies in diagnosis and treatment [3,4,5]. There are several causes of dementia, including Alzheimer’s disease (AD), frontotemporal dementia (FTD), Lewy body dementia (LBD), and vascular dementia (VD) [6,7]. AD, the most prevalent form of dementia, is characterized by the accumulation of beta-amyloid plaques and tau tangles in the brain, which disrupt neural connectivity and cause memory loss and progressive cognitive decline [8,9]. FTD is characterized by prominent behavioral and personality changes, often associated with abnormal protein deposits in the frontal and temporal lobes [10,11]. Unlike AD, memory loss may not be an initial symptom and individuals with FTD may exhibit socially inappropriate behaviors and language difficulties [12]. LBD is characterized by the presence of Lewy bodies, abnormal protein deposits causing cognitive fluctuations, visual hallucinations, and motor symptoms resembling Parkinson’s disease (PD) [13,14]. LBD may also involve fluctuating alertness and attention, which distinguishes it from other forms of dementia [15,16]. Finally, VD results from impaired blood flow to the brain, typically caused by vascular pathology, including strokes [17,18]. Symptoms vary depending on the affected areas of the brain and may include difficulties with planning and organizing, as well as memory impairment [19]. VD is unique in its association with cardiovascular risk factors and cerebrovascular events [7]. Each subtype of dementia presents a distinct set of challenges. Accurate diagnosis and targeted interventions in the realm of dementia care require an understanding of the characteristics, symptoms, and differences between AD, FTD, LBD, and VD [20,21].

Dementia-related diseases can be caused by genetic regulators, particularly non-coding RNAs (ncRNAs) such as microRNA (miRNA), long non-coding RNA (lncRNA), and circular RNA (circRNA) [22,23]. MiRNA, a small ncRNA of about 20 nucleotides, regulates its target gene by binding complementarily to the 3′ untranslated region (UTR) of the gene [24,25]. One of the most well-known functions of miRNA is to inhibit the expression of its target gene through degradation or translation inhibition of target messenger RNA (mRNA) [26,27]. A mature miRNA is produced from a miRNA gene in several steps. First, the miRNA is transcribed from the miRNA gene to produce primary miRNA (pri-miRNA). This pri-miRNA is then processed into precursor miRNA (pre-miRNA) by the Drosha-DiGeorge Syndrome Critical Region Gene 8 (DGCR8) complex [28,29]. The pre-miRNA is transported to the cytoplasm by exportin 5, where it is cleaved by the Dicer-TAR RNA-binding Protein (TRBP) complex to form double-stranded RNA (dsRNA) [30]. The dsRNA is unwound to produce the mature single-stranded miRNA, which associates with Argonaute (AGO) proteins to form the RNA-induced silencing complex (RISC) [31,32,33]. When miRNA binds to target genes complementarily, it can be influenced by other ncRNAs with complementary sequences of miRNA [34]. These ncRNAs act as regulators of miRNA, with lncRNA and circRNA being prominent examples. LncRNA, characterized by its length exceeding 200 nucleotides, intricately modulates gene expression by acting as a molecular sponge, decoy, or guide for miRNA [35,36]. The biogenesis of lncRNA involves a series of steps for its functional maturation. LncRNA is transcribed by RNA polymerase II and subsequently undergoes several processes, including splicing to remove introns and ligate exons, as well as post-transcriptional modifications such as RNA editing and chemical modifications. Processed lncRNA exists in either the nucleus or the cytoplasm, where it is involved in various cellular functions [37,38,39]. Meanwhile, circRNA, distinguished by its covalently closed-loop structure, also contributes to the regulatory landscape as another crucial player [40]. Transcription of genes in the nucleus initiates RNA synthesis, using the DNA sequence as a template to make precursor RNA. A unique process called backsplicing is involved in the formation of circRNA, which differs from typical linear mRNA production. During backsplicing, a downstream splice donor site (5′ end) of the precursor RNA joins an upstream splice acceptor site (3′ end), making the RNA circular closed-loop structure with no 5′ or 3′ ends. This often involves skipping one or more exons, resulting in circRNAs with specific coding sequences. The size of the circRNA varies depending on which exons are included. The biogenesis of lncRNA involves a series of steps to ensure their functional maturation or exclusion [41,42,43]. CircRNA is known for its resistance to exonucleolytic degradation and function as miRNA sponge [24,44]. Therefore, these distinctive features of circRNA make them noteworthy in the field of genetic regulation.

**Figure 1:**
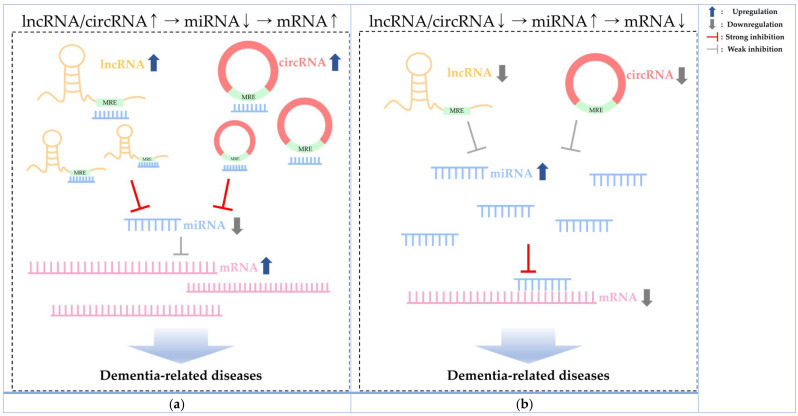
The mechanisms of dementia pathogenesis through ceRNA interactions. (**a**) Increased expression of lncRNA or circRNA acts as a miRNA sponge, inhibiting miRNA expression. It leads to the high expression of a target gene involved in dementia. (**b**) Downregulated expression of lncRNA or circRNA induces upregulation of miRNA expression, resulting in decreased mRNA expression levels of a target gene associated with dementia. MRE: miRNA response elements.

Recent studies have highlighted the significance of competing endogenous RNA (ceRNA), a complex network of RNA–RNA interactions, as promising targets for disease biomarkers and therapeutic interventions [45,46]. In particular, ceRNA includes regulatory elements such as lncRNA and circRNA, both acting as molecular competitors for miRNA binding. Furthermore, the intricate dynamics of ceRNA interactions have recently emerged as crucial orchestrators in the complex symphony of genetic expression [47,48]. The interactions between miRNAs and ceRNAs play an essential role in shaping the pathogenesis of dementia and hold promise as diagnostic indicators and therapeutic targets (Figure 1) [49,50]. Therefore, we discuss these interactions, focusing on the complex dynamics of ceRNAs, especially miRNAs, in dementia-related diseases and provide a comprehensive overview.

## 2. Dementia-Related Diseases and miRNAs

Dementia-related diseases are influenced by a multitude of genes that interact with miRNAs as regulatory elements [51]. By examining the findings of numerous previous studies, this section clarifies the role of miRNAs in the development and progression of dementia. Understanding the interaction of these factors is crucial for accurate diagnosis and personalized management to identify genetic markers and potential therapeutic targets within this complex landscape [52].

### 2.1. Alzheimer’s Disease and miRNA

AD is thought to be caused by a combination of genetic, age-related, and environmental factors, including the accumulation of abnormal brain proteins, neuroinflammation, vascular complications, and lifestyle choices [8,53]. Recent studies have shown that changes in gene expression, regulated by miRNAs, play an important role in AD [54,55,56]. While these miRNAs are typically present within cells, they are also secreted into extracellular fluids, influencing the pathophysiology of AD. Therefore, this subsection discusses the effects of miRNAs on AD, depending on their location.

#### 2.1.1. Differentially Expressed miRNAs in AD

It is widely recognized that miRNAs are ubiquitous in cells and influence gene expression. Accordingly, miRNAs stand out as a focal point of investigation, not only for their regulatory role in disease pathogenesis but also as promising candidates for diagnostic and therapeutic targets (Table 1).

The upregulated miRNAs in AD, including miR-20b-5p, miR-140, miR-425-5p, and miR-132, were identified to be implicated in neuronal protection by regulating cellular apoptosis and inflammatory responses [57,58,59,60]. The significance of amyloid-beta peptide (Aβ), derived from amyloid-beta precursor protein (APP) in AD pathogenesis, was highlighted by Wang et al. Their study elucidated the complex implications of miR-20b-5p in AD, noting changes in its levels in different brain regions during disease progression. In neuronal cells, elevated levels of miR-20b-5p were observed to decrease intracellular Ca^2+^ transients, leading to lower cell density through decreased synaptic branching, and ultimately reduce cortical thickness. Consequently, upregulation of miR-20b-5p was associated with an increased risk of AD. Paradoxically, miR-20b-5p was also found to reduce APP levels by binding to its mRNA, although the underlying mechanism remains unclear. This result highlighted the complex interplay between miR-20b-5p, *APP*, and the development of AD, which warrants further investigation into their interactions [57]. Another study revealed that overexpression of miR-425-5p increased tau phosphorylation and glycogen synthase kinase-3beta (GSK-3β), leading to increased cell death. These results were obtained by correlating miR-425-5p with heat shock protein B8 (*HSPB8*), which is known to play a role in the removal of misfolded proteins, thereby contributing to the regulation of neurodegenerative diseases. The authors suggested that miR-425-5p could be a potential therapeutic target for AD treatment [59].

A noteworthy feature of AD is characterized by many genetic factors, especially beta-amyloid plaques and tau tangles, which are associated with the Beta-Secretase 1 (*BACE1*) gene and specific mutations with miRNA interactions [93]. For this reason, several miRNAs, including miR-103, miR-107, miR-149, miR-9-5p, and miR-29c-3p, which decreased in AD, were observed to target *BACE1* [75,78,79,82]. These downregulations were linked to enhanced neuronal protection and growth by attenuating Aβ signaling through *BACE1*, thus emphasizing the potential therapeutic significance of miRNAs in AD. Recent research further supported the role of miRNA dysfunction in AD pathology, especially in increased Aβ production and impaired clearance [73]. In addition, miR-31, identified as decreasing in AD patients, has been found to simultaneously reduce *APP* and *BACE1* mRNA levels, leading to significant improvements in memory deficits and cognitive inflexibility. Overexpression of miR-31 also attenuated AD neuropathology, suggesting that miR-31 modulation of *APP* and *BACE1* could offer a promising therapeutic approach for AD [83]. On the other hand, there is another study carried out with a genetic factor regulated by miRNAs other than *BACE1*. Downregulated miR-326 reduced tau phosphorylation and prevented neuronal apoptosis by binding to its target gene Vav Guanine Nucleotide Exchange Factor 1 (*VAV1*) in the JNK signaling pathway. This finding suggested that miR-326 has potential as a target for AD treatment, improving cognitive function and inhibiting neuronal apoptosis *VAV1* [72].

#### 2.1.2. Exosomal miRNA and G-Quadruplex Structure in AD

Exosomal miRNAs (exo-miRs) refer to miRNAs that are released into extracellular fluids after being encapsulated within exosomes, extracellular vesicles [94,95]. Exosomes released by diverse cells play a crucial role in intercellular communication by conveying miRNAs and other substances [96,97]. Exosomes are considered carriers of miRNAs and are thought to play a critical role in the understanding, diagnosis, and treatment of AD, like cellular miRNAs [98,99,100]. The diagnostic process for AD involves a comprehensive evaluation that integrates clinical, neuropsychological, imaging, and laboratory assessments to differentiate between its various subtypes [101,102,103]. Accurate diagnosis of AD is essential, and while tissue analysis is crucial, the risks involved highlight the growing need for non-invasive detection methods. Exosomes are poised to meet this demand, particularly for early diagnosis, by utilizing differentially expressed exosomal miRNAs as key diagnostic tools. For example, subjective cognitive decline (SCD), one of the earliest symptoms of AD, is not only a symptom of memory loss, but is also highly genetically influenced, highlighting the importance of exosomal miRNAs for its early detection. The researchers suggested that peripheral neuronal-derived exosomal Aβ, tau, and upregulated exo-miR-384 were key modifiers in the pathogenesis of SCD as well as AD and could be used for early diagnosis of AD [67]. In another study, researchers demonstrated that overexpression of exo-miR-22 inhibited pyroptosis by targeting gasdermin D (*GSDMD*), thereby enhancing memory and motor abilities in AD through the suppression of inflammation. Given the therapeutic potential of exosomes for AD, the researchers hypothesized that exo-miR-22 could have a positive impact on AD outcomes [87].

Meanwhile, there is a structural study of the relationship between miRNA and AD. The G-quadruplex, a structure found in nucleic acids with guanine-rich sequences, is known to play a crucial role in protein binding and is thought to be a target for several diseases [104,105,106]. One study showed that the AD-associated single nucleotide polymorphism (SNP), rs2291418, located within pre-miR-1229, led to the formation of a G-quadruplex structure in equilibrium with the hairpin structure. In individuals with the rs2291418 in pre-miR-1229, there was an increase in the mature form of pre-miR-1229, miR-1229-3p, in AD cases. The miR-1229-3p bound complementarily to sortilin-related receptor 1 (*SORL1*), which is involved in the processing and trafficking of Aβ. The study demonstrated that pre-miR-1229 has not only a typical hairpin but also a G-quadruplex secondary structure, which leads to the production of a large amount of miR-1229-3p and a decrease in the expression of *SORL1*. The identification of a G-quadruplex structure within pre-miR-1229 represents a promising avenue for therapeutic intervention in AD [68].

### 2.2. Other Dementia-Related Diseases and miRNA

Although AD is the most commonly recognized cause of dementia, there are several other types of dementia such as FTD, LBD, and VD [107]. Due to the low incidence of these dementia-related diseases excluding AD, research on the role of miRNAs is limited compared to AD [108]. Nonetheless, some studies have explored genetic factors and miRNA regulation in these other types of dementia (Table 2). The current research landscape reveals a significant gap in our understanding of the mechanisms and treatment options for FTD, LBD, and VD when compared to AD [109]. Therefore, urgent attention is required to conduct further studies and investigations into the underlying mechanisms and therapeutic strategies in FTD, LBD, and VD. Such efforts have the potential to expand our knowledge base and ultimately improve patient outcomes in these complex neurological disorders.

#### 2.2.1. Differentially Expressed miRNAs in FTD

First of all, FTD is a neurodegenerative disorder characterized by progressive damage to the frontal and temporal lobes, with genetic factors being recognized as major contributors to its onset [127]. Several publications have focused on identifying miRNAs targeting these genetic factors in the quest for potential therapeutic interventions. Upregulated miRNAs like miR-29b and miR-659-3p were representatively target granulin precursor (*GRN*) which is known to be involved in FTD progression [110,111]. Jiao et al. confirmed that the absence of progranulin (PGRN), which is encoded by *GRN*, is implicated in certain types of FTD, a significant neurodegenerative disease with early onset. The mechanisms governing PGRN expression are not well-understood. The researchers found that ectopic expression of miR-29b reduced PGRN levels, and induced progranulin deficiency, leading to FTD. Also, they suggested that targeting miR-29b or other miRNAs could be a novel therapeutic approach to increase PGRN levels in individuals with FTD, as a post-transcriptional regulator of PGRN [110]. On the other hand, MiR-632, known to be associated with mutant *GRN*, but limited in functional studies, showed diagnostic potential for the disease. Significantly downregulated in the cerebrospinal fluid (CSF) of FTD patients compared to healthy controls, the decreased expression of miR-632 suggests its utility as a valuable diagnostic biomarker for both genetic and sporadic forms of FTD, distinguishing them from AD and healthy individuals [112].

#### 2.2.2. Differentially Expressed miRNAs in LBD

LBD is characterized by the presence of abnormal protein deposits called Lewy bodies in the brain, leading to progressive cognitive decline, visual hallucinations, and motor symptoms resembling PD [128]. However, it differs from PD in several ways [129,130,131,132]. In LBD, cognitive impairment and visual hallucinations often precede or occur alongside motor symptoms, whereas PD primarily exhibits motor symptoms like tremors, stiffness, and bradykinesia, with cognitive decline emerging later in the disease progression [129]. The distinct temporal presentation of symptoms sets these two conditions apart despite their shared feature of Lewy bodies. Differences in genetic factors, such as the alpha-synuclein (*SNCA*) gene, also play distinct roles in PD and LBD [130,133]. Therefore, there is a need to identify key markers, such as miRNAs, to differentiate LBD from PD in diagnosis. While research on miRNAs in LBD is scarce, there is a study aimed at distinguishing LBD from PD based on miRNA expression patterns. The researchers explained that it is uncertain whether miR-7-5p directly decreases *SNCA* expression, but higher levels of miR-7-5p may contribute to maintaining baseline *SNCA* mRNA expression. Additionally, the short structural variant rs777296100-poly-T in *SNCA*, which may affect miRNA binding, was moderately associated with LBD but not with PD. This research also suggested that the miR-7/*SNCA* axis may be a potential diagnostic marker for LBD, as it is significantly upregulated in LBD compared to normal [116].

#### 2.2.3. Differentially Expressed miRNAs in VD

Finally, VD is characterized by cognitive decline resulting from impaired blood flow to the brain, often arising as the second most common cause of dementia after AD [134]. Unlike other dementia-related diseases, VD is associated with cerebrovascular disease and vascular-related brain damage, leading to disruptions in cognitive function [135]. The key distinguishing feature of VD is its vascular etiology, where strokes or other vascular events contribute to the onset and progression of VD [136]. Therefore, understanding the underlying vascular factors is crucial for accurate diagnosis and targeted interventions in cases of VD. In this regard, research on miRNAs in VD offers promise for understanding complex genetic factors and exploring the potential for diagnosis and treatment. For example, miR-210-5p, miR-134, miR-150, and miR-181a are upregulated in VD, resulting in synaptic loss and impairing spatial learning, memory functions, speech, and language, which also contribute to associated neurodevelopmental disorders [117,118,120]. For example, one study aimed to investigate the impact of miRNAs on early VD. MiR-210-5p was increased in the hippocampus of rats exposed to 4 weeks of ischemia and binds complementarily to synaptosomal-associated protein of 25 KDa (*Snap25*) mRNA, causing cognitive deficits and synaptic loss, suggesting a potential new therapeutic avenue for the treatment of VD [117]. Forkhead box P2 (*Foxp2*), which is inhibited by miR-134-5p, is a transcription factor crucial for vocal learning. Elevated miR-134-5p levels were observed in the cortex of VD model rats, antagomir of miR-134-5p significantly mitigated synaptic protein loss by upregulating *Foxp2*. This implicated the miR-134-5p/*Foxp2* axis in early VD-related cognitive decline [118]. Furthermore, miR-150 was investigated for the impacts on VD. The results suggested that reducing miR-150 levels may alleviate VD symptoms, significantly improving cognitive impairment and reducing neuron apoptosis in the brain by upregulating homeobox A1 (*HOXA1*) expression [120]. Additionally, miR-134 was found to decrease Cofilin 2 levels, thereby regulating oxidative stress and autophagy in the brain. Suppression of miR-134 in VD rats improved cognitive function by modulating oxidative stress and autophagy, resulting in induced levels of Cofilin 2 [121]. Another study revealed that downregulated miR-181a can increase PTEN-induced kinase 1 (*PINK1*)/Parkin expression and enhanced mitophagy to improve mitochondrial function and enhance cognitive capabilities in VD. These findings highlight the potential of miR-181a modulation to attenuate cognitive decline by regulating mitophagy processes in VD [123].

Conversely, downregulated miRNAs such as miR-132-3p, miR-322-5p, and miR-181a were associated with inflammatory responses and cognitive improvement in VD [123,125,126]. For instance, one study assessed the levels of miR-132-3p using a VD mouse model induced by bilateral carotid artery occlusion. The overexpression of miR-132-3p led to the downregulation of RAS P21 Protein Activator 1 (*RASA1*) expression that influenced the Ras/Akt/GSK-3β pathway, leading to improved neuronal survival and neurite outgrowth [125]. In addition, miR-322-5p was found to alleviate the development of VD by targeting tetraspanin 5 (*TSPAN5*), which may play a critical role in modulating cognitive function and mitophagy in the context of VD. *TSPAN5*, a member of the tetraspanin family, is known for its involvement in cell signaling, growth, and motility. Studies have implicated it in VD pathology, including activation of *ADAM10* and the Notch pathway. In experimental models of neuronal injury and cognitive dysfunction, *TSPAN5* expression was elevated and its overexpression attenuated the effects of miR-322-5p on neuronal injury. Conversely, the knockdown of *TSPAN5* alleviated cognitive dysfunction in rats subjected to cerebral ischemia. This is a valuable finding that may provide a potential regulatory mechanism for studying cognitive function [126]. These downregulated miRNAs are considered potential treatments for VD, highlighting the importance of studying genetic factors of VD.

## 3. Dementia-Related Diseases and Other Non-Coding RNAs

The significance of ceRNA and its relevance to dementia has garnered increasing attention in the scientific community, particularly as it has been revealed that ceRNA interactions involving lncRNA or circRNA, miRNA, and mRNA play a critical role in the regulation of gene expression [137,138,139]. In the context of dementia, understanding these intricate ceRNA networks holds promise for unraveling the molecular mechanisms underlying the disease [140,141,142,143]. Investigating how these RNA molecules interact and influence each other can provide valuable insights into potential diagnostic markers and therapeutic targets for dementia [144]. The exploration of ceRNA networks may contribute to advancing our comprehension of the complex RNA-mediated regulatory pathways implicated in dementia pathogenesis.

### 3.1. LncRNA

**Figure 2:**
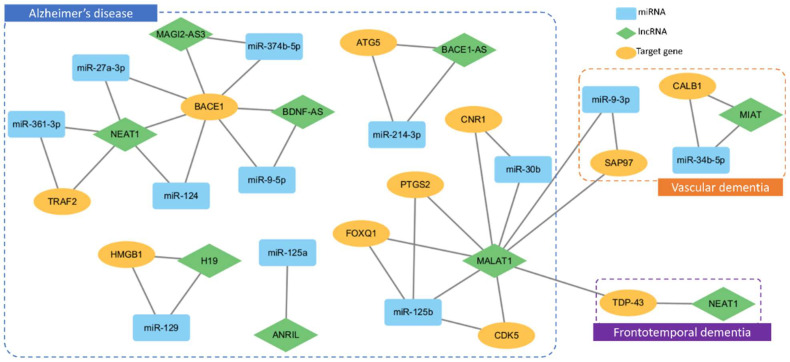
The complex interactions observed among miRNAs (light blue rectangles), lncRNAs (green rhombuses), and target genes (yellow ovals) in the context of AD with 10 interactions denoted by a blue dotted box, FTD with 1 interaction marked by a purple dotted box, and VD with 2 interactions identified within an orange dotted box. The interactions reflect the regulatory roles of miRNAs in inhibiting target genes, while lncRNAs act as miRNA sponges, thereby suppressing the inhibitory activity of miRNAs and contributing to the intricate network of gene expression regulation in dementia-related disorders.

LncRNAs are a diverse group of non-protein-coding transcripts that exhibit distinct features. They play a significant role in regulating gene expression through various mechanisms, including acting as sponges for miRNAs and functioning as ceRNAs [145,146,147,148]. In this capacity, lncRNAs possess miRNA response elements, allowing them to sequester and competitively inhibit miRNA activity. This role enables lncRNAs to modulate the availability of miRNAs for their target mRNAs, thereby influencing post-transcriptional gene expression regulation [149,150]. Understanding these distinctive features and the involvement of lncRNAs in miRNA sponging mechanisms contributes to unraveling complex regulatory networks. Importantly, these mechanisms have implications for various biological contexts, including their potential relevance to dementia [151]. In particular, some studies have explored the roles of lncRNAs Metastasis Associated Lung Adenocarcinoma Transcript 1 (*MALAT1*), also known as non-coding Nuclear-Enriched Abundant Transcript (*NEAT*) 2, and *NEAT1* in dementia [152,153]. The role of these lncRNAs in dementia is expected to involve complex regulatory functions [154]. These findings contribute to a growing body of research exploring the functions of *MALAT1*, *NEAT1*, and various other lncRNAs in the context of dementia-related diseases (Figure 2, Table S1).

Some research has been conducted on lncRNAs, sponging miRNAs that target *BACE1*, a key gene involved in AD, which accounts for about 80% of dementia cases [155]. MiR-374b-5p, miR-9-5p, and miR-27a-3p were representative examples and one of the studies investigated the regulatory role between lncRNA, Membrane Associated Guanylate Kinase, WW, and PDZ Domain Containing 2 Antisense RNA 3 (*MAGI2-AS3*), and miR-374b-5p in Aβ-induced neurotoxicity and neuroinflammation in AD [156,157,158,159]. MiR-374b-5p targets *BACE1* and *MAGI2-AS3* functions as a sponge for miR-374b-5p. Upon exposure to Aβ, there was an increase in *MAGI2-AS3* and a decrease in miR-374b-5p levels in neuronal and microglial cells. Reduction of *MAGI2-AS3* and overexpression of miR-374b-5p alleviated neurotoxicity and neuroinflammation, while miR-374b-5p inhibition reverses these effects. Serum levels of *MAGI2-AS3* and miR-374b-5p in Alzheimer’s patients were negatively correlated and associated with disease severity, suggesting the *MAGI2-AS3*/miR-374b-5p axis as a potential biomarker and therapeutic target for AD [156]. Another study highlights the role of Brain-Derived Neurotrophic Factor-AS (*BDNF-AS*) in the pathogenesis of AD by demonstrating its elevated expression in the peripheral blood of AD patients, correlating with cognitive decline. Elevated *BDNF-AS* promoted neurotoxicity by enhancing *BACE1* expression through competitive binding with miR-9-5p, facilitating amyloid deposition and cognitive impairment in AD mice. Additionally, miR-27a-3p and *NEAT1* showed consistent expression trends in AD patients, with decreased levels of miR-27a-3p and increased *NEAT1* in serum correlating with disease severity and Aβ deposition, suggesting their involvement in AD progression [158].

In FTD, transactivation response DNA binding protein 43 kDa (TDP-43) is thought to be one of the causes of disease onset, along with *GRN*. Although TDP-43 is known to be a nuclear RNA-binding protein, its targeting and processing of mRNA are not well-understood [160]. Studies have predicted that mislocalized TDP-43 contributes to neurodegeneration in FTD by regulating the stability of mRNA such as *GRN* [161]. One study revealed that in the brain of FTD patients, TDP-43 was significantly increased and that TDP-43-regulated mRNAs largely control neural development or encode proteins associated with neurological disorders. Also, RNA-binding analysis results showed that TDP-43 tended to bind to the lncRNAs *MALAT1* and *NEAT1*. Highlighting the importance of TDP-43 in regulating mRNAs through lncRNAs, understanding the interaction of TDP-43 with RNA is crucial for insights into the neurodegenerative processes of FTD [162].

VD, the second leading cause of dementia, also involves mechanisms related to lncRNA [163]. Exosomes containing the lncRNA Myocardial Infarction-Associated Transcript (*MIAT*) can improve cognitive function in VD by enhancing hippocampal pathology, reducing oxidative stress, and downregulating inflammatory and amyloid-beta-related markers. The therapeutic effects of *MIAT* were implicated with miR-34b-5p [164]. On the other hand, elevated levels of *MALAT1* in VD were found to correlate with deficits in spatial learning and memory, as well as a reduction in miR-9-3p. In addition, synapse-associated protein-97 (*SAP97*) was found to be upregulated in the hippocampus of those with VD. In vitro experiments on hippocampal neurons showed that miR-9-3p negatively regulated *SAP97* expression. Furthermore, the downregulation of *MALAT1* increased miR-9-3p and reduced *SAP97*, whereas inhibition of miR-9-3p rescued the reduction in *SAP97*. This study suggests that *MALAT1* upregulated *SAP97* by targeting miR-9-3p in the hippocampus of VD mice, shedding light on the molecular mechanisms underlying VD [165].

While investigations into lncRNA have been conducted in various dementia-related disorders, the majority of research has been concentrated on AD. However, the available studies are limited and there is a noticeable bias towards AD. Moreover, no significant research on lncRNA in the context of LBD has been identified. lncRNAs have potential as useful diagnostic markers and therapeutic targets in diseases; further extensive research is needed to improve our understanding of these conditions [155,166,167].

### 3.2. CircRNA

**Figure 3:**
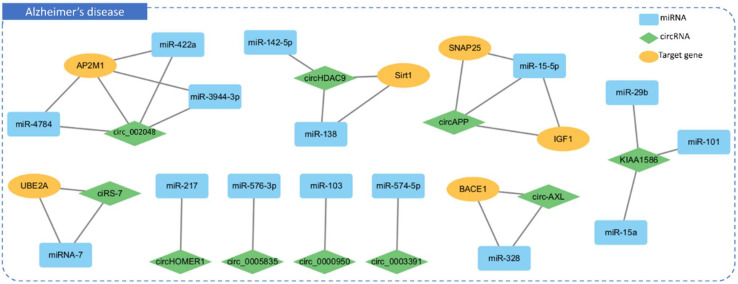
The intricate interactions among miRNAs (light blue rectangles), circRNAs (green rhombuses), and target genes (yellow ovals) in AD (blue dotted box) with 10 interactions. The figure demonstrates miRNAs’ regulatory roles in inhibiting target genes, alongside circRNAs functioning as miRNA sponges to suppress miRNA activity, thus forming a complex gene expression network in AD.

CircRNAs are used as versatile regulators due to their unique characteristics, such as resistance to degradation, stability, and widespread presence across species [168,169,170]. They exhibit tissue-specific expression patterns and dynamic regulation during various physiological and pathological processes [171,172]. Functionally, circRNAs act as miRNA sponges, interact with RNA-binding proteins, and influence alternative splicing [168]. Several studies in AD have investigated the association between circRNAs and miRNAs, yet similar research in other dementia-related disorders is limited (Figure 3, Table S2) [171,173,174,175,176,177].

For instance, downregulation of hsa_circ_002048 was found to upregulate three miRNAs (hsa-miR-422a, hsa-miR-4784, and hsa-miR-3944-3p), potentially limiting the expression of adaptor-related protein complex 2 subunit mu 1 (*AP2M1*). This dysregulation led to impaired endocytosis, including autophagy, resulting in elevated proinflammatory cytokines and neurotoxic β-amyloid levels. These cascading events contributed to inflammation, β-amyloid deposition, and tau hyperphosphorylation, impacting AD progression. While offering insights into AD pathogenesis, this study suggested that these ncRNAs could serve as potential targets for diagnostic biomarkers, paving the way for advancements in AD diagnosis and treatment [178]. Another study focused on identifying dysregulated circRNA, hsa_circ_0003391, in the peripheral blood of AD patients and its association with clinical manifestations. Hsa_circ_0003391 was specifically downregulated in AD patients compared to other dementia types. The study demonstrated a significant reduction in hsa_circ_0003391 expression in the peripheral blood of AD patients, correlating with the clinical characteristics of AD. This study identified hsa_circ_0003391 as a potential biomarker for AD diagnosis and suggested insights for improving diagnostic approaches. Additionally, the research offered a new perspective for investigating the pathogenesis of AD, potentially leading to the development of innovative therapies targeting ncRNA [179]. In a specific case related to *BACE1*, a study aimed to investigate the influence of circ-AXL on neuronal injury and inflammation in cellular models of AD, and to elucidate the underlying molecular mechanisms. The study revealed that overexpression of circ-AXL resulted in increased apoptosis, reduced neurite outgrowth, and elevated levels of inflammatory cytokines in cellular AD models. Circ-AXL was found to negatively regulate miR-328 and positively modulate *BACE1* expression. Conversely, miR-328 had a negative regulatory effect on *BACE1*. Overexpression of miR-328 reduced apoptosis, promoted neurite outgrowth, and decreased inflammatory cytokine levels in cellular AD models, while knockdown of miR-328 had the opposite effect. Notably, the attenuation of miR-328 reduced the impact of circ-AXL knockdown on cellular AD models. Furthermore, upregulation of *BACE1* exacerbated neuronal injury and inflammation, counteracting the effects of miR-328 overexpression in cellular AD models. These findings suggested that circ-AXL may be a promising therapeutic target in AD due to its regulation of *BACE1* through miR-328 [180].

Despite the unique characteristics and functional roles of circRNAs, research on their involvement in other forms of dementia, excluding AD, is limited. Therefore, there is a significant gap in understanding circRNA involvement in other dementia-related diseases. Thorough investigations into the features and functions of circRNAs in various dementia-related conditions are necessary to comprehend their role in neurodegenerative processes fully. Such research is crucial for uncovering the potential diagnostic, prognostic, and therapeutic implications of circRNAs, advancing dementia research beyond AD.

## 4. Dementia Pathogenesis with Genetic Factors and ncRNA Insights

**Figure 4:**
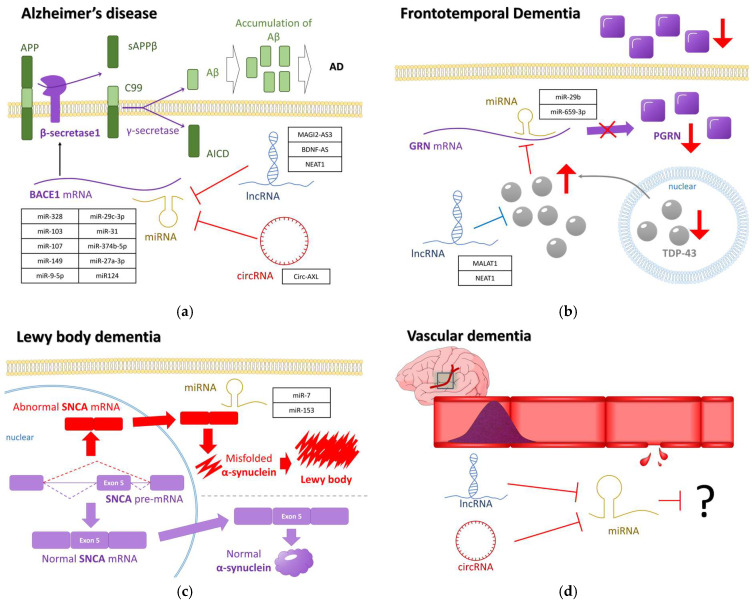
The key molecules in dementia-related diseases. (**a**) AD primarily results from the accumulation of Aβ in cells due to increased expression of *BACE1*. Although miRNAs can complementarily bind to *BACE1* mRNA, lncRNAs and circRNAs may act as miRNA sponges instead. (**b**) FTD arises from mislocalization of TDP-43, normally located in the nucleus, leading to increased expression in the cytoplasm. This cytoplasmic TDP-43 suppresses *GRN* expression, potentially regulated by downregulated miRNAs and lncRNAs. (**c**) LBD is associated with Lewy bodies formed by misfolded α-synuclein. (**d**) The specific genetic factors involved in VD, potentially due to vascular damage, are not precisely known, although multiple genetic factors are suggested to be involved. AD: Alzheimer’s disease; APP: Amyloid-beta precursor protein; BACE1: beta-Secretase; MAGI2-AS3: Membrane Associated Guanylate Kinase, WW, and PDZ Domain Containing 2 Antisense RNA 3; BDNF-AS: Brain-Derived Neurotrophic Factor-AS; NEAT1: Nuclear-Enriched Abundant Transcript; FTD: frontotemporal dementia; GRN: granulin precursor; PGRN: progranulin; MALAT1: Metastasis Associated Lung Adenocarcinoma Transcript 1; TDP-43: Transactivation response DNA binding protein 43 kDa; LBD: Lewy body dementia; SNCA: alpha-synuclein; VD: vascular dementia.

The study aimed to give a comprehensive overview of the involvement of diverse genetic factors in dementia-related diseases and to explore their correlations with genes and ncRNAs. In particular, the relationship between key regulators in each dementia-related disease was visualized (Figure 4). Specifically in AD, overexpression of *BACE1* leading to Aβ accumulation was regarded as a primary cause. In addition, the regulation of miRNAs targeting *BACE1* and the role of lncRNAs and circRNAs as miRNA sponges were key regulatory factors in AD (Figure 4a) [53,93,157,180]. Research in FTD has primarily focused on the correlation between *GRN* and TDP-43, with limited simultaneous studies on their regulation by ncRNAs. Therefore, this study concentrated on predicting the correlation between mislocalized TDP-43, which regulates GRN, and related miRNAs and lncRNAs (Figure 4b) [110,111,162]. LBD is characterized by misfolded α-synuclein due to splicing errors, but there have been few studies on miRNA involvement. Therefore, this study focused on the only miRNA research about miR-7 and miR-153, which regulate exon-skipped *SNCA* mRNA binding and are expected to inhibit LBD (Figure 4c) [116]. Finally, VD is linked to vascular damage with various implicated genetic factors. However, research on the key regulators that mainly function in pathogenesis is limited. Therefore, we tried to envision the need for interaction to identify these key regulators concerned with ncRNAs (Figure 4d) [181].

## 5. Conclusions

Dementia, which includes a broad category of neurocognitive disorders characterized by progressive decline, indicates several diseases such as AD, FTD, LBD, and VD. Although numerous factors contribute to the development of dementia-related diseases, genetic factors regulated by ncRNAs play a critical role in their pathogenesis. While many studies have been conducted on how ncRNAs affect dementia in animal models, there are limitations as these models do not fully mimic human conditions. In addition, although there are some studies using brain tissue from patients, it is difficult to obtain living brain tissue, and the tissue used in research is generally severely damaged, reducing the accuracy of the research. To address this issue, recent studies have focused on ncRNAs present in serum, which is relatively easier to obtain and does not require surgical procedures. Nevertheless, there is still a lack of research on ncRNAs involved in the signaling pathways associated with the development of dementia-related diseases. Therefore, mechanistic studies on ncRNAs in dementia-related diseases are necessary. The accumulation of these studies could ultimately contribute to the development of effective diagnostic and therapeutic strategies for dementia by identifying clinically important ncRNAs and elucidating their mechanisms.

## Figures and Tables

**Table 1:** List of differentially expressed miRNAs related to AD.

miRNA Expression in AD	miRNA	Target Gene	Clinical Value	Function	Study Model	Reference
UP	miR-20b-5p	*APP*	N/A	Reduce intracellular Ca^2+^ transients during neuronal membrane depolarization	Human tissues and in vitro	[57]
	miR-140	*PINK1*	Biomarker and therapeutic target	Improve amyloid pathology and mitochondrial dysfunction while inhibiting cellular autophagy	In vivo	[58]
	miR-425-5p	*HSPB8*	Therapeutic target	Reduce cell apoptosis and tau phosphorylation	Human tissues and in vitro	[59]
	miR-10b-5p	*HOXD10*	Therapeutic target	Reduce nerve cell apoptosis, inflammatory response, and oxidative stress	In vivo	[60]
	miR-455-5p	*CPEB1*	Therapeutic target	Synaptic plasticity and memory disorders	In vivo	[61]
	miR-592	*KIAA0319*	Therapeutic target	Promotion of oxidative stress injury in astrocytes	In vivo and in vitro	[62]
	miR-1273g-3p	*TIMM13*	Biomarker and therapeutic target	Mitochondrial dysfunction	Human tissues and in vitro	[63]
	miR-485-3p	*CD36*	Biomarker and therapeutic target	Inhibit microglial Aβ phagocytosis	In vivo and in vitro	[64]
	miR-134-5p	*CREB-1*	Therapeutic target	Regulation of long-term plasticity and cellular correlation	In vivo and in vitro	[65]
		*BDNF*				
	miR-384	-	Diagnostic biomarker	Involved in immune response	Human samples	[66]
	miR-1229-3p	*SORL1*	Therapeutic target	Engaged in the processing and movement of Aβ	In vitro	[67]
	miR-29a-3p	*C1QTNF6*	Diagnostic biomarker	Migration of neurons and evolution of the nervous system	Human samples	[68]
		*ROBO1*				
		*DAAM2*				
	let-7i-5p	-	Diagnostic biomarker	Regulating APP and BACE1, leading to AD pathology	Human samples	[69]
	miR-15a-5p					
	miR-34a	*VAMP2*	Therapeutic target	Abnormalities in energy metabolism, resting state network activity, and synaptic plasticity	Human tissues and in vivo	[70]
		*SYT1*				
DOWN	miR-92a-3p	*SYNJ1*	Diagnostic biomarker	Connected to protein and lipid pathways, transcription, structural function, and amyloid-beta clearance/cell signaling	Human samples	[68]
		*CBLN4*				
		*BCL2L2*				
		*NEFH*				
		*REST*				
	miR-132	*FOXA1*	Therapeutic target	Improve cognitive impairment	In vivo	[71]
	miR-326	*VAV1*	Therapeutic target	Tau phosphorylation and neuronal apoptosis	In vivo	[72]
	miR-146a-5p	*Nkd2*	Therapeutic target	Inhibit LPS/Aβ-induced neuroinflammation and regulate a microglial phenotype	In vivo and in vitro	[73]
	miR-195	*ApoE4*	Therapeutic target	Regulate tau hyperphosphorylation and secretion	Human tissues and in vivo	[74]
	miR-103	*BACE1*	Prognosis biomarker	Enhance neurite outgrowth and reduce neuronal apoptosis	Human tissues	[75]
	miR-107					
	miR-455-3p	-	N/A	Improve neuronal activity and overall brain function	In vivo	[76]
	miR-146a	*TRAF6*	N/A	Suppress astrocyte inflammation in AD	In vivo	[77]
	miR-149	*BACE1*	Diagnostic biomarker	Reduce accumulation of Aβ and improve the viability of neurons	Human serum and in vitro	[78]
	miR-9-5p	*BACE1*	Diagnostic biomarker	Regulate differentiation of post-mitotic neurons from neural progenitor cells	Human serum	[79]
		*SIRT1*				
	miR-107	*FGF7*	N/A	Ameliorate Aβ-induced inflammation and apoptosis	Human serum and in vitro	[80]
	miR-23b	*GnT-III*	Therapeutic target	Inhibit oxidative stress and activate the Akt/GSK-3β signaling pathway	In vivo and in vitro	[81]
	miR-29c-3p	*BACE1*	Therapeutic target	Aβ-induced suppression of neuronal viability and increase in apoptosis	In vivo	[82]
	miR-31	*APP*	Therapeutic target	Improve cognitive function and prevent the progression of the disease	In vivo and in vitro	[83]
		*BACE1*				
	miR-212	*PDCD4*	N/A	Mitigate Aβ25-35 induced neurotoxicity through modulation of the PI3K/AKT pathway	Human serum and in vitro	[84]
	miR-22-3p	*SOX9*	Therapeutic target	Enhance apoptosis attenuation and reduce Aβ accumulation	In vivo and in vitro	[85]
	miR-126a-3p	*EFHD2*	Therapeutic target	Consolidate contextual fear memory	In vivo and in vitro	[86]
	miR-22	*GSDMD*	Therapeutic target	Enhancing memory and motor abilities	In vivo	[87]
	miR-92a-3p	*MAPT*	Diagnostic biomarker	Control the expression of tau in a neuroblastoma	Human samples and in vitro	[88]
	miR-320a					
	miR-132	*MAPT*	Therapeutic target	Partially recovered tau metabolism and memory	Human tissues, in vivo and in vitro	[89]
	miR-212					
	miR-188-3p	*BACE1*	Therapeutic target	Enhanced synaptic and cognitive function through decreased neuroinflammation and Aβ due to MAGL inhibition	In vivo and in vitro	[90]
	miR-132-3p	*FOXO1a*	N/A	Hyperphosphorylation of tau	Human tissues	[91]
	miR-512	*cFLIP*	N/A	Apoptosis initiating factor, APAF-1 activity, activated caspase-3, elevated caspase-4 and caspase-8, and the TUNEL assay was negative in the regions where neurons displayed hyperphosphorylated tau	Human samples	[92]
		*MCL1*				

N/A: not available.

**Table 2:** List of differentially expressed miRNAs involved in other dementia-related diseases.

Name of Disease	miRNA Expression in Disease	miRNA	Target Gene	Clinical Value	Function	Study Model	Reference
FTD	UP	miR-29b	*GRN*	Therapeutic target	Induce progranulin deficiency and development of neurodegenerative diseases	In vitro	[110]
		miR-659-3p	*GRN*	N/A	Neurotrophic, anti-inflammatory activity and act as neuroprotective against oxygen/glucose deprivation, oxidative injury, and hypoxia stress	In vivo and in vitro	[111]
	DOWN	miR-632	*GRN*	Diagnostic biomarker	Prevents apoptosis, averting degenerative changes in the frontal and temporal lobes	Human samples	[112]
		miR-132	*TMEM106B*	Therapeutic target	Disrupt endosomal-lysosomal pathways, trapping PGRN in *TMEM106B*-positive compartments, elevating intracellular PGRN levels	Human tissues and in vitro	[113]
		miR-212					
		miR-124	*CHMP2B*	Therapeutic target	Reduce AMPAR levels and partial rescue of behavioral deficits	Human tissues and in vivo	[114]
		miR-127-3p	-	Diagnostic biomarker	Regulate neuronal differentiation	Human samples	[115]
LBD	UP	miR-7	*SNCA*	N/A	The process of forming dopamine-producing neurons	Human tissues and in vitro	[116]
		miR-153					
VD	UP	miR-210-5p	*Snap25*	Therapeutic target	Synaptic loss impacting spatial learning and memory	In vivo	[117]
		miR-134-5p	*Foxp2*	Therapeutic target	Contribute to speech and language and associated neurodevelopmental disorders	In vivo and in vitro	[118]
		miR-93	*TSPAN5*	Therapeutic target	Reduce inflammation and promote positive regulation of the TLR4 signaling pathway	In vivo	[119]
		miR-150	*HOXA1*	Biomarker and therapeutic target	Increase cell apoptosis	In vivo and in vitro	[120]
		miR-134	*Cofilin 2*	N/A	Regulate oxidative stress and autophagy	In vivo	[121]
		miR-154-5p	*PRKAA2*	Biomarker and therapeutic target	Impair EPC function and angiogenesis	Human samples and in vivo	[122]
		miR-181a	*PINK1*	N/A	Alleviate mitochondrial dysfunction and improve cognitive function	In vivo	[123]
			*Parkin*				
	DOWN	miR-216a	*RSK2*	Therapeutic target	Regulate oxidative stress and neuroinflammation	In vivo	[124]
		miR-132-3p	*RASA1*	Therapeutic target	Improve neuronal and synaptic dysfunction by activating the Ras/Akt/GSK-3β pathway	In vivo	[125]
		miR-322-5p	*TSPAN5*	Therapeutic target	Improve cell apoptosis, inflammatory response, and cognitive function	In vivo and in vitro	[126]

N/A: not available.

## Data Availability

Not applicable.

## Supplementary Materials

The supporting information can be downloaded at: https://www.mdpi.com/article/10.3390/ijms25116190/s1. References [20,156,157,158,159,162,164,165,172,173,174,175,176,177,178,179,180,182,183,184,185,186,187,188] are cited in the Supplementary Materials.
